# Matched analysis of circulating selenium with the breast cancer selenotranscriptome: a multicentre prospective study

**DOI:** 10.1186/s12967-023-04502-y

**Published:** 2023-09-23

**Authors:** Kamil Demircan, Ylva Bengtsson, Thilo Samson Chillon, Johan Vallon-Christersson, Qian Sun, Christer Larsson, Martin Malmberg, Lao H. Saal, Lisa Rydén, Åke Borg, Jonas Manjer, Lutz Schomburg

**Affiliations:** 1 Institute for Experimental Endocrinology, Cardiovascular-Metabolic-Renal (CMR)-Research Center, Charité-Universitätsmedizin Berlin, Freie Universität Berlin, Humboldt-Universität zu Berlin, and Berlin Institute of Health, Berlin, Germany; 2 Berlin Institute of Health (BIH), Biomedical Innovation Academy (BIA), Berlin, Germany; 3 Department of Surgery, Skåne University Hospital Malmö, Lund University, Malmö, Sweden; 4 Division of Oncology, Department of Clinical Sciences Lund, Lund University, Lund, Sweden; 5 Division of Translational Cancer Research, Department of Laboratory Medicine, Lund University, Lund, Sweden; 6 Department of Oncology, Skåne University Hospital, Lund, Sweden

**Keywords:** Selenoproteins, SELENOP, Glutathione peroxidase, Thyroid hormones, Prognosis

## Abstract

**Introduction:**

Low serum selenium and altered tumour RNA expression of certain selenoproteins are associated with a poor breast cancer prognosis. Selenoprotein expression stringently depends on selenium availability, hence circulating selenium may interact with tumour selenoprotein expression. However, there is no matched analysis to date.

**Methods:**

This study included 1453 patients with newly diagnosed breast cancer from the multicentric prospective Sweden Cancerome Analysis Network – Breast study. Total serum selenium, selenoprotein P and glutathione peroxidase 3 were analysed at time of diagnosis. Bulk RNA-sequencing was conducted in matched tumour tissues. Fully adjusted Cox regression models with an interaction term were employed to detect dose-dependent interactions of circulating selenium with the associations of tumour selenoprotein mRNA expression and mortality.

**Results:**

237 deaths were recorded within ~ 9 years follow-up. All three serum selenium biomarkers correlated positively (p < 0.001). All selenoproteins except for GPX6 were expressed in tumour tissues. Single cell RNA-sequencing revealed a heterogeneous expression pattern in the tumour microenvironment. Circulating selenium correlated positively with tumour *SELENOW* and *SELENON* expression (p < 0.001). In fully adjusted models, the associations of *DIO1*, *DIO3* and *SELENOM* with mortality were dose-dependently modified by serum selenium (p < 0.001, p = 0.020, p = 0.038, respectively). With increasing selenium, *DIO1* and *SELENOM* associated with lower, whereas *DIO3* expression associated with higher mortality. Association of *DIO1* with lower mortality was only apparent in patients with high selenium [above median (70.36 µg/L)], and the HR (95%CI) for one-unit increase in log(FPKM + 1) was 0.70 (0.50–0.98).

**Conclusions:**

This first unbiased analysis of serum selenium with the breast cancer selenotranscriptome identified an effect-modification of selenium on the associations of *DIO1*, *SELENOM*, and *DIO3* with prognosis. Selenium substitution in patients with *DIO1*-expressing tumours merits consideration to improve survival.

**Supplementary Information:**

The online version contains supplementary material available at 10.1186/s12967-023-04502-y.

## Introduction

Breast cancer remains a significant global health challenge, with an estimated 2.3 million new cases and 685,000 deaths annually [1]. Discovery of novel prognostic factors may improve prognosis by identifying high risk women early and by personalizing or intensifying therapy regimens [2].

Recently, there has been growing scientific interest in the role of the essential trace element selenium (Se) in risk, progression and prognosis of breast cancer [3]. Several large epidemiological studies have reported an independent association of low dietary intake or marginal serum levels of Se with a distinctly poor prognosis [4–8]. Se affects various physiological pathways by being translationally incorporated into the products of 25 human selenoprotein genes, that mainly act in antioxidative defense systems, quality and structure control, and by activating or inactivating thyroid hormones [9–11]. The biosynthesis of selenoproteins depends on their RNA expression level and on Se availability, and hereby correlates with Se intake and serum Se concentrations [12]. Accordingly, marginal Se intake correlates to low serum concentrations of the Se transporter selenoprotein P (SELENOP), resulting in suboptimal systemic Se supply [13]. This deficit is e.g. translated in kidney into relatively low biosynthesis and secretion, and hence lower activity of the plasma glutathione peroxidase 3 (GPx3). Accordingly, low serum Se and SELENOP concentrations as well as low serum GPx3 activity have been associated with higher risk of mortality and recurrence after breast cancer diagnosis [4]. At the same time, tumour tissue gene expression levels of several selenoproteins, such as iodothyronine deiodinases (DIO), glutathione peroxidases (GPx) or thioredoxin-reductases (TXNRD) have also been reported as prognostic factors for breast cancer in large-scale genomic profiling studies [14–16].

Despite the evidence that both circulating Se and tumour gene expression of selenoproteins are associated with breast cancer prognosis, there is a lack of data from a matched analysis of serum Se biomarkers and tumour selenoprotein expression. Se availability constitutes a key factor for RNA stability of certain selenoprotein transcripts, and for translation, i.e., Se controls the rate of biosynthesis of the different selenoproteins from a given RNA expression level [17, 18]. Therefore, the association between selenoprotein transcript expression and breast cancer prognosis may be modified by Se availability, and a sufficiently high Se status may be required for translating differences in RNA expression levels into the corresponding gene products and disease-modifying selenoprotein activities.

To test this hypothesis, we simultaneously analysed three complementary biomarkers of Se status (total serum Se, SELENOP, GPx3) and conducted bulk RNA-sequencing of tumours of 1453 patients with a new diagnosis of primary invasive breast cancer. The patients were followed for ~ 9 years. The main aim was to assess whether the association between tumour RNA expression of selenoprotein genes with breast cancer prognosis is modified by circulating Se and selenoprotein levels.

## Methods

### SCAN-B study design

The Sweden Cancerome Analysis Network Breast Study (SCAN-B) is a multicentre population based prospective study that intends to identify novel biomarkers for early identification of patients with a poor prognosis based on serum and tumour tissue (genomics) as a matrix. In brief, the study, which is still ongoing since 2010 enrols patients with a newly diagnosed or suspected primary breast cancer in the catchment area of Southern Sweden at multiple cancer centres. The study was approved by the Regional Ethical Review Board in Lund, Sweden (Registration numbers 2009/658, 2010/383, 2012/58, 2013/459, and 2015/277) and registered under the ClinicalTrials.gov ID NCT02306096. All enrolled participants have given written informed consent for inclusion in the study and analyses/procedures, which were conducted as an integrative part of clinical routine, as described before [19].

### Follow up and covariate assessment

All clinical data was collected by standardized procedures by clinicians and referred to the Swedish National Quality Registry for Breast Cancer (NKBC). Vital status was obtained from the Swedish National Population Registry, which maintains records for all Swedish citizens. Covariates included patients’ characteristics, tumour characteristics, and information on treatment procedures, as described before [5, 19]. These variables comprised age, sex, and information on menopausal status of the patients was reported. Histopathological information comprised tumour size, the side of involved breast, histopathological subtype, histological grade (Nottingham Histological Grade, NHG), Ki67 expression, HER2 expression, ER expression, PGR expression, and number of involved lymph nodes. Information on therapy comprised the surgical procedure regarding the breast, regarding the axillary lymph nodes, application of endocrine or immune therapy, radiation or chemotherapy. Mode of diagnosis, i.e. either clinical or by screening was also reported. The almost full completeness (99.9%) and over 90% validity of NKBC with regard to information on vital status, and other have been externally validated before [20].

### Serum selenium biomarkers

Laboratory analyses and results for quantification of Se biomarkers in this study have been described before [4, 5]. In brief, total Se was measured using total reflection X-ray fluorescence (TXRF) spectroscopy, SELENOP was measured using a validated commercial ELISA (selenOtest, selenOmed GmbH, Berlin, Germany), and activity of glutathione peroxidase 3 was measured using an established coupled enzyme reaction. All analyses were conducted by scientists and technicians blinded to any clinical information, with samples arranged in a randomized order with regard to order of enrolment in the study, as reported before [4, 5].

### Selenoprotein gene expression in patient’s tumours

The detailed protocols for RNA-sequencing have been described before, i.e. either the protocol as shown before [19] or using the Illumina stranded TruSeq mRNA procedure. Protocols were established using the Illumina NeoPrep system or the KingFisher system. For the purpose of this study, gene expression values in Fragments Per Kilobase per Million reads (FPKM) was generated. An established analysis pipeline was used to extract FPKM values by alignment and estimation of gene expression data. The pipeline has been described in detail before [21], and involves the tools picard tools, trimmomatic, bowtie, hisat2, stringtie, dbSNP56 and GENCODE. Genes were annotated based on gene and transcript definitions contained in GENCODE Release 27. After adding an offset of 1, FPKM data were log-transformed for the analyses in this study. Single cell RNA sequencing data was accessed from the Single Cell Portal of the Broad Institute (https://singlecell.broadinstitute.org/single_cell), and included 100,064 single cells from 26 primary breast cancer tumours [22]. Access and visualization was conducted on March 21st 2023.

### Statistical analyses

Descriptive statistics were presented as median along with interquartile range (IQR) for continuous variables, and as frequencies along with percent for categorical data. Correlation matrices were generated to depict the relationship between RNA expression of selenoproteins and serum Se biomarkers. Non-parametric Spearman’s rank correlation test was applied to compute Spearman’s R and p values for the correlations, and cut-off for p-values were adjusted in correlation matrices taking into account the number of genes tested (0.05:23).

Linear multivariable Cox proportional hazards models were employed to calculate hazard ratios along with 95% confidence intervals (CI) for one increment in log(FPKM + 1) in gene expression for each gene. Models were adjusted for established clinical predictors of breast cancer prognosis, i.e. age at diagnosis (years), menopausal status (pre-menopausal, post-menopausal, uncertain), tumour size (mm), NHG (I, II, III), lymph node involvement (at least 4, 1 to 3, submicrometastasis (< 0.2 mm) or no involvement), HER2 expression (positive, negative), ER expression (positive, negative), PGR expression (positive, negative), histological type (ductal, lobular, ductal + lobular/other, other), laterality (right or left breast). Regression analyses were conducted in the entire cohort, and separately in the low and high Se group, based on each different biomarker, while median level of the cohort served as unbiased cut-off. In order to detect potential effect modification by Se biomarkers, an interaction term with the continuous Se biomarker variable was added. An interaction was considered statistically significant in case of *p*_interaction_ < 0.05. Significant interactions between continuous variables were visualized using contour plots, and by visualizing the association based on tertiles of Se biomarker. Missing variables made up only a small portion (0.4%) of all values contained in variables in the adjusted models (Additional file 1: Fig. S1), therefore Cox regression models were computed using complete cases.

All analyses were conducted using the R language (The R Foundation for Statistical Computing, version 4.3.0) on the RStudio environment (RStudio, PBC, version 2022.2.3.492).

## Results

Based on availability of tissue and RNA-sequencing data, as well as serum sampling and Se status assessment, a total of 1453 patients with complete RNA-sequencing and data on serum Se biomarkers were included in the final analyses. A detailed description of the study flow chart is included in Additional file 1: Fig. S2. Follow-up time comprised 9,701 years in total, corresponding to a mean follow-up time of 6.68 years, and 237 deaths were recorded in this time frame.

### Baseline patient and tumour characteristics

Baseline patient characteristics and tumour characteristics as well as applied therapy regimens according to vital status during the study are presented in Table 1. Patients were divided based on whether they died during the follow-up period or survived. Patients that died over the course of the follow-up were older at time of diagnosis, more frequently post-menopausal, had larger tumours, more lymph nodes involved, lower serum Se and SELENOP concentrations and a lower serum GPx3 activity. The association of serum Se biomarkers with prognosis have been assessed in this cohort previously, displaying dose-dependent associations of low serum Se with a poor prognosis, independent of various confounders [5]. Table 2 depicts the mode of clinical diagnosis, and the treatment regimens applied in the study cohort, comprising both adjuvant and surgical methods.

**Table 1:** Baseline patients characteristics according to vital status

Characteristic	Alive, n = 1216	Dead, n = 237
Age	63 (52, 69)	73 (65, 82)
Menopausal status		
Post-menopausal	896 (74%)	215 (91%)
Pre-menopausal	258 (21%)	18 (7.6%)
Uncertain	56 (4.6%)	3 (1.3%)
( Missing)	6	1
Laterality		
Left	618 (51%)	135 (57%)
Right	598 (49%)	102 (43%)
Size (mm)	17 (12, 22)	22 (15, 31)
(Missing)	7	2
Number of lymph nodes involved		
≥ 4	102 (8.7%)	38 (17%)
1–3	336 (29%)	46 (20%)
No involvement	708 (60%)	136 (60%)
Submicrometastasis	25 (2.1%)	6 (2.7%)
(Missing)	45	11
Nottingham histological grade		
I	212 (18%)	24 (10%)
II	569 (48%)	95 (41%)
III	414 (35%)	114 (49%)
(Missing)	21	4
Ki67 expression		
High	163 (56%)	27 (71%)
Low	128 (44%)	11 (29%)
(Missing)	925	199
Histological type		
Ductal	999 (82%)	187 (79%)
Lobular	146 (12%)	30 (13%)
Other	49 (4.0%)	18 (7.6%)
Ductal + lobular/other	20 (1.6%)	2 (0.8%)
(Missing)	2	0
HER2 expression		
Negative	1,041 (86%)	202 (86%)
Positive	164 (14%)	33 (14%)
(Missing)	11	2
ER expression		
Negative	158 (13%)	61 (26%)
Positive	1,056 (87%)	176 (74%)
(Missing)	2	0
PGR expression		
Negative	321 (26%)	99 (42%)
Positive	893 (74%)	138 (58%)
(Missing)	2	0
Selenium (µg/L)	72 (62, 82)	63 (52, 74)
SELENOP (mg/L)	4.10 (3.34, 4.90)	3.71 (2.75, 4.49)
GPx3 (U/L)	208 (177, 240)	189 (152, 229)

Median (IQR); n (%)

**Table 2:** Therapy regimens according to vital status

Characteristic	Alive, n = 1216	Dead, n = 237
Diagnosis		
Mammography	629 (52%)	74 (31%)
Other	570 (48%)	162 (69%)
(Missing)	17	1
Surgical procedure breast		
Mastectomy	476 (39%)	164 (69%)
Partial mastectomy	740 (61%)	73 (31%)
Surgical procedure axilla		
Clearence only	137 (11%)	49 (21%)
No axillary surgery	3 (0.2%)	4 (1.7%)
Sampling	14 (1.2%)	5 (2.1%)
Sentinel node + clearence	328 (27%)	41 (17%)
Sentinel node surgery	733 (60%)	137 (58%)
(Missing)	1	1
Endocrine therapy	976 (80%)	162 (69%)
(Missing)	1	2
Chemotherapy	486 (40%)	64 (27%)
(Missing)	1	2
Immunotherapy	148 (12%)	17 (7.2%)
(Missing)	1	2
Radiotherapy	826 (68%)	107 (46%)
(Missing)	1	2

n (%)

### Serum selenium and tumour selenoprotein expression

**Fig. 1:**
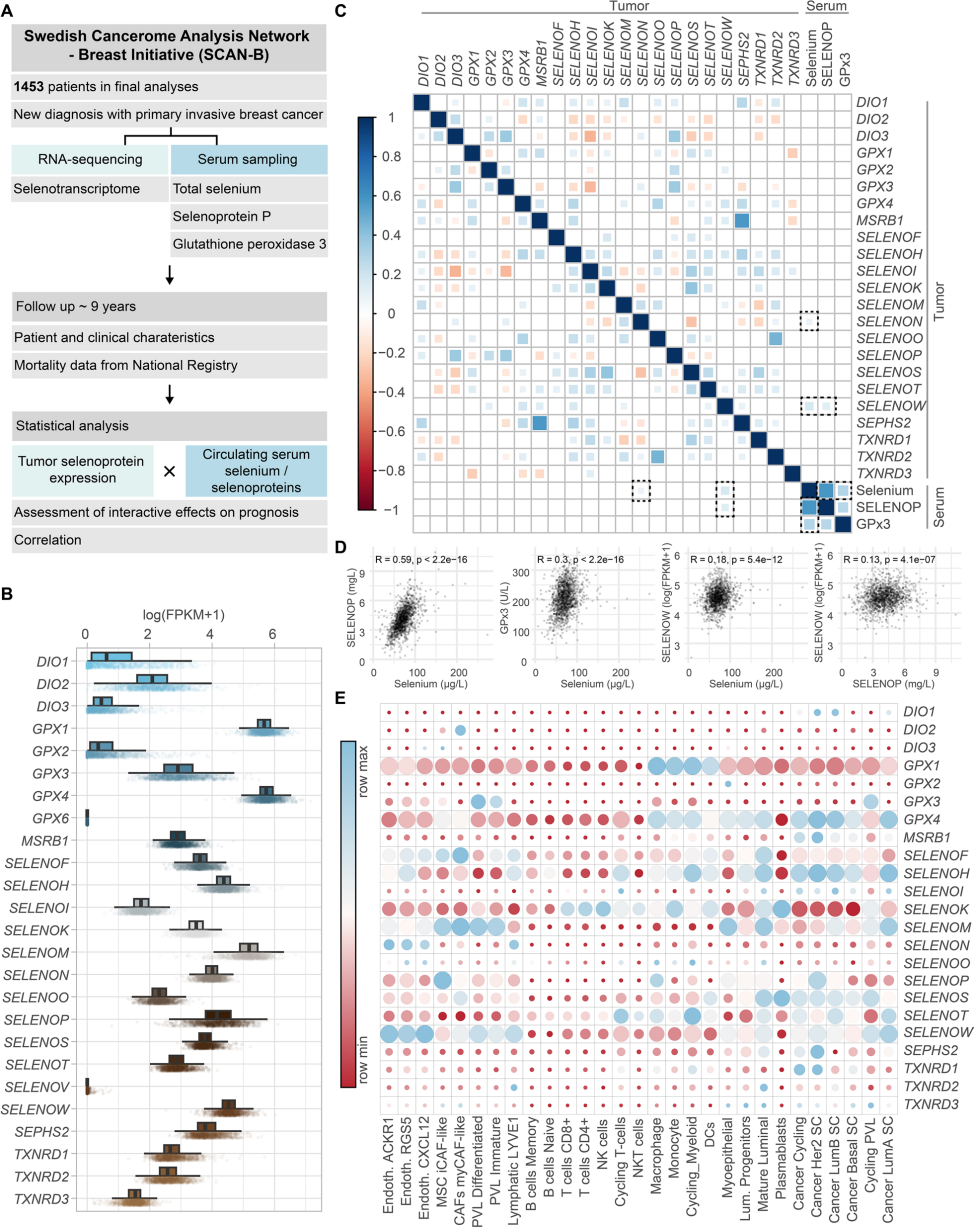
** A** Study scheme. **B** Gene expression of selenoproteins in tumour samples of 1453 patients. **C** Spearman’s correlation matrix of tumour gene expression of selenoprotein genes and circulating selenium biomarker concentrations. **D** Spearman’s correlation of serum biomarkers with each other and serum biomarkers with SELENOW expression in the tumour. **E** Single cell RNA-expression of selenoprotein genes in different cells in breast cancer

Figure 1 A depicts the study design. Selenoprotein mRNA expression levels within the tumours are displayed in Fig. 1B. As *GPX6* was not expressed in the tumour and *SELENOV* displayed only a low expression, both genes were excluded from further analyses. Analyses included the deiodinase family involved in thyroid hormone regulation (*DIO1-3)*, the glutathione peroxidases involved in antioxidative defence (*GPX1-4*), thioredoxin reductases involved in cellular redox regulation (*TXNRD1-3)*, selenoproteins located within the endoplasmic reticulum (e.g. *SELENOM*, *SELENOF* etc.), as well as the other selenoproteins with specific functions [9]. Figure 1 C displays the correlation between tumour selenoprotein expression and serum Se and selenoprotein levels. Within the group of selenoprotein genes, the highest correlation was between *MSRB1* and *SEPHS2* (R = 0.57), while *SELENOI* and *DIO3* displayed the most prominent negative correlation (R = -0.34). All three serum biomarkers correlated positively, with serum Se and SELENOP displaying the highest correlation (R = 0.59). Serum Se biomarkers were mostly not correlated with selenoprotein gene expression levels, except for a weak correlation between serum Se and SELENOP with *SELENOW* (R = 0.18 and R = 0.13, respectively) (Fig. 1D), and between serum Se and *SELENON* (R = 0.082). Figure 1E displays the selenoprotein gene expression in different cells in breast cancer.

### Selenoprotein gene expression and survival based on selenium biomarkers

**Fig. 2:**
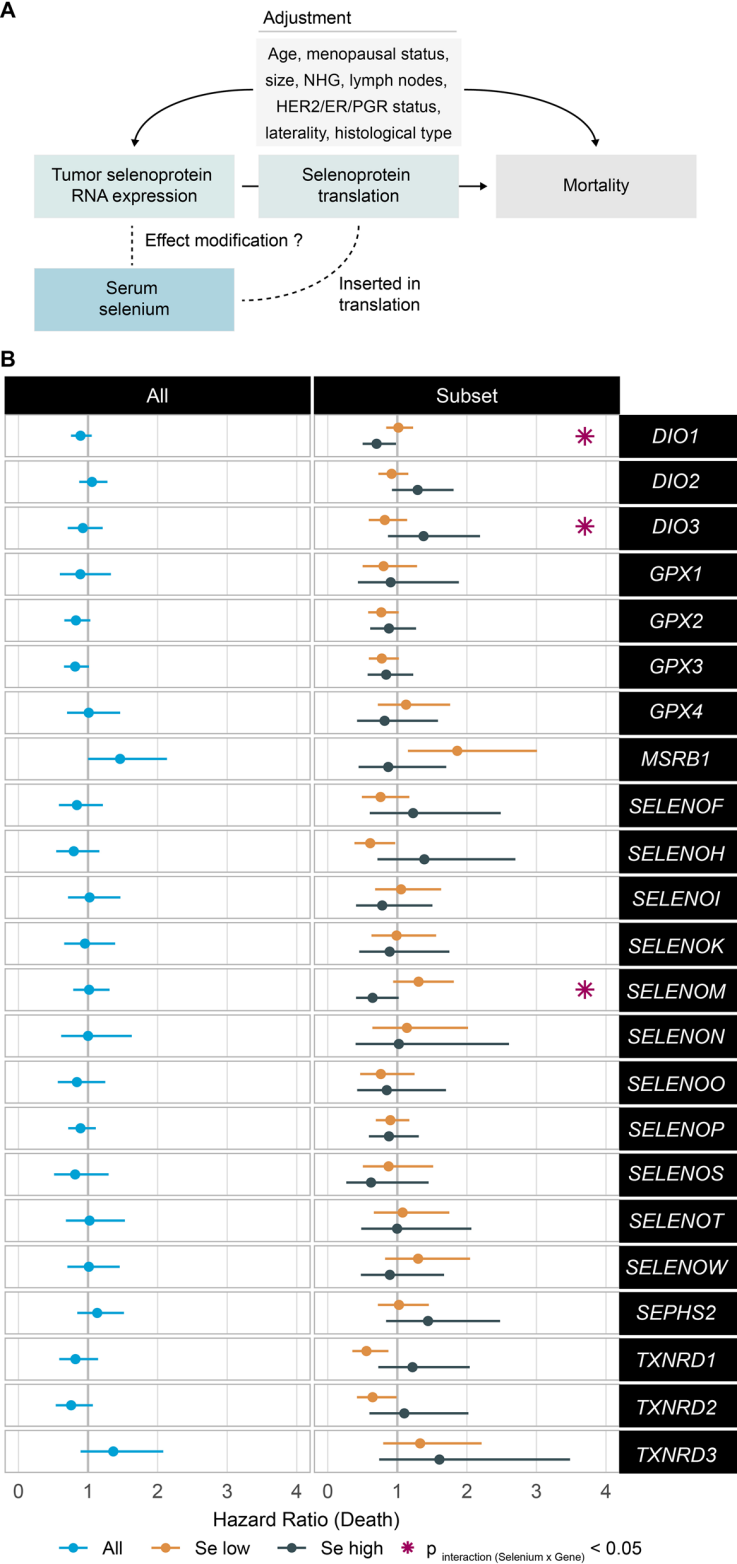
** A** Analysis scheme. **B** Cox regression models in the whole cohort and in low and high selenium subsets, divided according to median selenium concentration of the cohort, i.e. 70.36 µg/L. All models were adjusted for age, tumour size, histological grade, lymph node involvement, expression of HER2/ER/PGR-Receptor, laterality of the tumour, and histological type. P for interaction was tested by adding an interaction term between serum selenium and the gene of interest, marked by purple asterisk

Figure 2 displays the association of selenoprotein mRNA expression of each gene with survival, in the whole cohort and in patients with low or high serum Se levels. There were significant interactions between serum Se with *DIO1*, *DIO3*, and *SELENOM*, p < 0.001, p = 0.020, and p = 0.038, respectively. Association of *DIO1* with lower mortality was only apparent in patients with high Se [above median (70.36 µg/L)], HR (95%CI) for one-unit increase in log(FPKM + 1) was 0.70 (0.50–0.98).

**Fig. 3:**
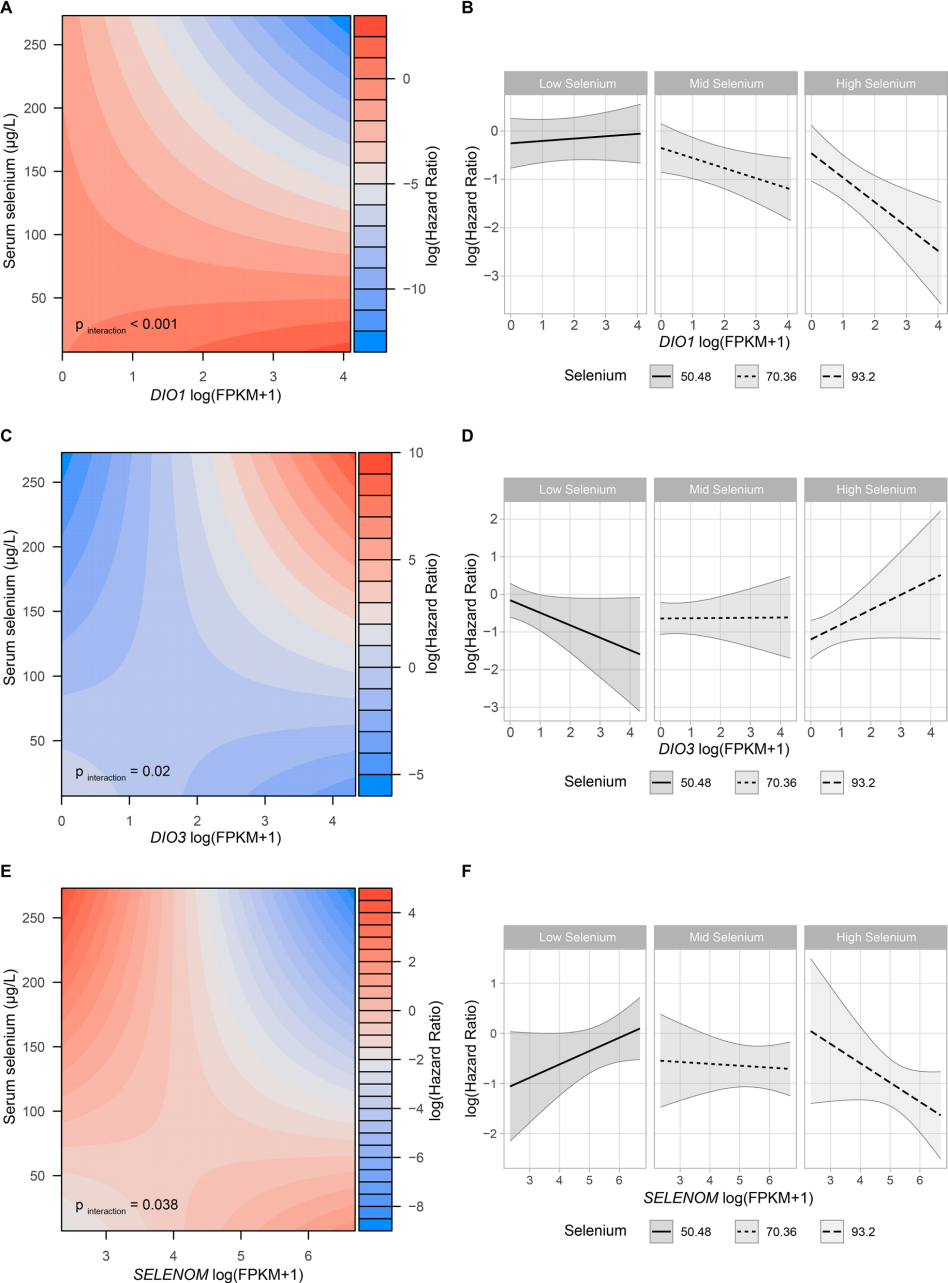
** A** Contour plot of the interaction between *DIO1* expression and serum selenium concentrations on mortality. **B** Cox regression models depicting the association of DIO1 expression with mortality according to 10th, 50th and 90th quantiles of circulating selenium. **C** Contour plot of the interaction between *DIO3* expression and serum selenium concentrations on mortality. **D** Cox regression models depicting the association of DIO3 expression with mortality according to 10th, 50th and 90th quantiles of circulating selenium. **E** Contour plot of the interaction between *SELENOM* expression and serum selenium concentrations on mortality. **F** Cox regression models depicting the association of *SELENOM* expression with mortality according to 10th, 50th and 90th quantiles of circulating selenium. All models were adjusted for age, tumour size, histological grade, lymph node involvement, expression of HER2/ER/PGR-Receptor, laterality of the tumour, and histological type

The complex interaction between serum Se and *DIO1*, *DIO3 and SELENOM* is depicted in Fig. 3. Figure 3 A displays lower hazard ratios for a simultaneous increase in serum Se and *DIO1* expression. This relationship is emphasized in Fig. 3B, which shows a decreased hazard ratio with increasing *DIO1* levels, however only in patients with a relatively high Se level, i.e. residing in the 2nd or 3rd tertile. On the other hand, Fig. 3C displays an inverse interaction of Se with *DIO3* levels, where increasing serum Se and simultaneous increase in *DIO3* are associated with an elevated hazard ratio. Accordingly, *DIO3* is associated with higher mortality in patients with high Se only, i.e., solely in the 3rd tertile (Fig. 3D). The interaction of serum Se with *SELENOM* was similar to *DIO1*, where *SELENOM* associated with a lower mortality rate with increasing Se levels (Fig. 3E and F).

Interaction analyses were conducted for serum SELENOP concentrations (Additional file 1: Fig. S3) and GPx3 activity levels (Additional file 1: Fig. S4). The interaction with *DIO1* remained prominent with the Se transporter SELENOP (p_*interaction*_ = 0.001). In patients with relatively high serum SELENOP, i.e. above the cohort median of 4.05 mg/L, HR for one-unit increase in log(FPKM + 1) for *DIO1* was 0.64 (0.48–0.86). There was no interaction between serum GPx3 activity and *DIO1*, *DIO3*, or *SELENOM* expression, but for *GPX1* RNA.

In further sensitivity analyses, interactions of serum Se with DIO1, DIO3 and SELENOM were tested after further adjusting for treatment methods used. Endocrine therapy, immune-, chemo- and radiotherapy as well as surgical procedures regarding the breast and axilla were added to the fully adjusted models one by one, and all combined, and nearly no changes were observed in p values for interaction (Additional file 1: Table S1).

An analysis of The Cancer Genome Atlas Program (TCGA) data displayed no overall associations of *DIO1*, *DIO3*, *SELENOM*, *SELENOW*, and *SELENON* with survival, when not incorporating serum Se (Additional file 1: Fig. S5), highlighting the need for consideration of both serum Se and tumour selenoprotein expression in order to ascertain effects of selenoprotein mRNA expression on prognosis.

## Discussion

In this large multicentric prospective study, the first matched analysis of circulating serum Se levels with the breast cancer selenotranscriptome was performed. As expected, serum Se levels were mostly unrelated to selenoprotein mRNA expression levels. Serum Se dose-dependently interacted with the association of *DIO1*, *DIO3*, and *SELENOM* and survival. With increasing serum Se, *DIO1* and *SELENOM* associated with lower, and *DIO3* expression associated with higher mortality. These opposing Se-dependent effects of *DIO1* and *DIO3* imply a mechanism of action involving alteration of local thyroid hormone status, in agreement with prior data and experiments with model systems [23, 24]. Selenium substitution particularly in patients with *DIO1* expressing tumours may improve survival, which should be considered for testing as an adjuvant therapy in randomized controlled trials.

Aberrations in the genome of breast cancer lead to alterations in expression of various genes, and hence to alterations in protein levels. Some of these proteins, such as HER2, ER, PGR are involved in key-regulatory mechanisms of breast cancer progression [25, 26]. Therefore, assessment of RNA-expression as indirect measure of actual protein levels constitutes an increasingly popular tool to predict prognosis [27–29]. For most proteins, RNA-expression has been shown to be a reliable proxy for actual protein levels [27]. Expression of selenoproteins however, is subject to more complex regulation involving a key limiting Se-dependent step in the translational process [30]. The incorporation of the characteristic Sec residue(s) via recoding of the UGA stop codon to a sense codon is an important regulator of translation during the biosynthesis of functional selenoproteins, which is mainly regulated by both transcript abundance and dietary intake of Se [31, 32]. Thus, RNA-sequencing of selenoproteins may reliably reflect true protein expression only in patients where Se intake is sufficiently high, ensuring saturated levels of circulating Se needed for optimal supply of tissues and high intracellular Se concentrations. In statistical terms, this hypothesis is described as a testable interaction between serum Se and gene expression levels in relation to survival.

In line with this hypothesis, we observed strong interaction effects, particularly for *DIO1* and *DIO3*. Deiodinases are the most important regulators of thyroid hormone activity, which is essential for cellular proliferation and differentiation, and hence implicated in cancer progression and cancer mortality [14, 33, 34]. Conflictingly, both increased and decreased circulating thyroid hormone levels have been linked to breast cancer survival, which may be attributed to the complex nature of thyroid hormone transport, metabolism and action [35–38]. Local regulators, such as thyroid hormone transporters, receptors and deiodinases play a critical role in thyroid hormone metabolism and action. Recent studies have demonstrated that DIO3 is a prognostic factor in breast cancer [14], and that e.g. low thyroid hormone receptor alpha 2 expression is associated with higher breast cancer mortality [35]. In addition to promoting proliferation, thyroid hormone action also mediates cellular differentiation in physiological processes [39]. Active thyroid hormones have been found to induce differentiation into a more benign phenotype in hepatic cancer cells [40]. In murine models of basal cell carcinoma, *Dio3* knockdown with concomitant increase in local T3 led to a five-fold decrease in tumour growth [41]. Our findings are in agreement with these studies, and suggest a Se-mediated potentiation of the favourable effects of DIO1 and the unfavourable effects of DIO3 on breast cancer survival. These findings indicate potential involvement of local thyroid hormone action in breast cancer progression, as DIO3 is the primary thyroid hormone inactivating enzyme, and DIO1 plays a crucial role in the deiodination of T4 to T3 [33]. Further epidemiological and mechanistic studies are necessary to investigate this hypothesis in more detail.

The association of SELENOM with mortality was also modified by circulating Se levels, potentiating the favourable association with survival. *SELENOM* encodes selenoprotein M, which is located in the endoplasmic reticulum and involved in protein folding [42]. Although little is known about the specific roles of selenoprotein M, its functional homolog selenoprotein F (SELENOF) has been shown to be involved in cancer progression [43, 44]. In line with our findings of favourable effects of *SELENOM* on survival, SELENOF was recently described as a tumour suppressor in breast cancer, and enhancing its expression reduced tumour growth both in vivo and in murine breast cancer models [45]. Similar to SELENOF, and in line with our findings higher expression of SELENOM has been shown to be a protective prognostic factor in other cancer types, such as gastric cancer and cholangiocarcinoma [46, 47].

Interactions with circulating Se were observed for three of the 23 tested selenoprotein genes only. One likely explanation for this finding is based on the organ- and selenoprotein-specific hierarchical regulation of selenoprotein expression, which serves to provide regular functioning of essential tissues in a Se deficient state. Hence, some of the selenoproteins are preferentially sustained in case of Se deficiency, while others display a more responsive decrease in activity and reduced expression when the supply is low [48]. Although this hierarchy is further regulated specific to different tissues, DIO1 has been shown to be one of the most responsive selenoproteins in the liver, displaying 95% decrease in activity in rats with severe Se deficiency [49]. This is in line with our findings, as the interaction between *DIO1* and circulating Se levels was the most prominent. Another explanation is the heterogenous distribution of selenoprotein gene expression within cells residing in the tumour and its microenvironment, as outlined in Fig. 1E. While *DIO1* as an instance is mostly abundant in cancer cells, *DIO2* shows nearly no expression in malignant cells, but rather in cancer associated fibroblasts, which may explain the lack of interaction for this member of the deiodinase family in our study.

Previously in the cohort used in this study, serum Se, serum SELENOP concentrations, activity of the serum GPx3 as well as novel autoantibodies targeting SELENOP were shown to be independent predictors of survival after breast cancer diagnosis, outperforming established clinical prognostic markers [4, 5]. Other large breast cancer studies are in line with these findings [6–8], and the effects seem to be also consistent for some other cancer entities [50–54]. In this study, we aimed to further exploring the potential mechanisms of action underlining the consistent associations, and to examine whether patients with tumours displaying certain selenoprotein gene expression profiles are particularly likely to benefit from a higher Se status. Our results indicate a potential mechanism of action in local thyroid hormone action due to significant and inverse interactions with *DIO1* and the thyroid hormone inactivating *DIO3*. Clinically, our results indicate that Se-deficient patients with *DIO1* expressing tumours may distinctly benefit from Se substitution, whereas patients with high DIO3 may not.

To the best of our knowledge, this study represents the first attempt to investigate the relationship between serum Se levels and RNA levels of selenoproteins in tumour tissues simultaneously in relation to survival, providing a more comprehensive understanding of the complex relationship between Se, selenoproteins, and cancer progression. Another noteworthy strength includes the large sample size as well as the large number of covariates assessed by physicians and pathologists. This allowed for conducting complex interaction analyses that require large sample sizes. The nearly complete database enabled studying effects independent of established clinical prognostic factors. The primary outcome, all-cause mortality derives from the Swedish National Registry, and the covariates were extracted from NKBC, which exhibited over 99.9% completeness and over 90% validity in an independent validation study conducted at time of participant recruitment of this study [20]. SCAN-B has been fully integrated into clinical routine, without interfering with clinical decision making, which increases generalizability of the results. Importantly, Se status was measured by multiple biomarkers, all linearly correlating, which ensures a correct quantification of the main exposure. All Se measurements were made in a double-blinded fashion, reducing risk of bias and increasing internal validity of the results.

Despite the strengths, a significant limitation is the observational nature, which precludes the ability to infer causality. Although adjustment was done for most important potential confounders, there may be residual confounding. A set of patients were excluded due to missing variables, although missingness was very low (Additional file 1: Fig. S1). The study’s population sample from Sweden may limit its generalizability to other populations and ethnicities other than European. Further studies in other populations are necessary to confirm the findings.

## Conclusion

Our unbiased analysis of circulating Se levels and the breast cancer selenotranscriptome revealed that Se modifies (potentiates) the associations of type 1 deiodinase expression with a favourable prognosis. On a mechanistical aspect, the study contributes to a growing evidence of an importance of thyroid hormones on cancer progression. Specifically, the favourable effects of *DIO1* and opposing effects of *DIO3* together argue for a beneficial effect of in-tissue local thyroid hormone action.

Clinically, the results provide a translational value, as serum Se measurement and (histo)pathological assessment of *DIO1* expression in tumours of breast cancer patients undergoing surgery can be integrated into clinical routine to stratify patients according to potential benefit from Se substitution. As our data provides observational evidence, however, this potential improved survival remains to be tested in well-designed RCTs. Nevertheless, this approach can readily be used in clinical routine in order to provide (surrogate) prognostic value.

### Supplementary Information

**Additional file 1: Figure S1. **Missing values in variables used in Cox regression models. **Figure S2. **Study flow chart. Adapted from Demircan K, Bengtsson Y, Sun Q, Brange A, Vallon-Christersson J, Rijntjes E, Malmberg M, Saal LH, Rydén L, Borg Å, Manjer J, Schomburg L. Serum selenium, selenoprotein P and glutathione peroxidase 3 as predictors of mortality and recurrence following breast cancer diagnosis: A multicentre cohort study. Redox Biol. 2021 Nov;47:102145. **Figure S3. **Cox regression models in the whole cohort and in low and high SELENOP subgroups. Subgroups were divided according to median SELENOP concentration of the cohort, i.e. 4.05 mg/L. All models were adjusted for age, tumour size, histological grade, lymph node involvement, expression of HER2/ER/PGR-Receptor, laterality of the tumour, and histological type. P for interaction was tested by adding an interaction term between serum SELENOP and the gene of interest, marked by purple asterisk. **Figure S4. **Cox regression models in the whole cohort and in low and high GPx3 subgroups. Subgroups were divided according to median GPx3 concentration of the cohort, i.e. 205 U/L. All models were adjusted for age, tumour size, histological grade, lymph node involvement, expression of HER2/ER/PGR-Receptor, laterality of the tumour, and histological type. P for interaction was tested by adding an interaction term between serum GPx3 and the gene of interest, marked by purple asterisk. **Figure S5.** Kaplan Meier analyses of genes interacting with serum selenium in TCGA-BRCA data. Patients were compared according to mRNA expression for each candidate gene, based on being in the highest quartile (Q4) vs lowest (Q1). Log-rank test was applied to detect differences. GEPIA2 was used to plot survival, accessed on 25th August 2023, on http://gepia2.cancer-pku.cn/). Tang, Z. et al. (2019) GEPIA2: an enhanced web server for large-scale expression profiling and interactive analysis. Nucleic Acids Res, 10.1093/nar/gkz430. **Table S1**. P values for interaction between DIO1, DIO3, SELENOM and serum selenium, further adjusted for therapy regimens. 

## Data Availability

RNA sequencing data are fully openly accessible at Mandalay Data [55]. Clinical and pathological tumour data are fully publicly available [21]. Data on trace elements can be applied for at the SCAN-B steering committee. R code for statistical analyses can be applied for from the corresponding authors.

## References

[1] Sung H, Ferlay J, Siegel RL, Laversanne M, Soerjomataram I, Jemal A (2021). Global Cancer Statistics 2020: GLOBOCAN estimates of incidence and mortality worldwide for 36 cancers in 185 countries. CA Cancer J Clin.

[2] Dowsett M, Dunbier AK (2008). Emerging biomarkers and new understanding of traditional markers in personalized therapy for breast cancer. Clin Cancer Res.

[3] Flowers B, Poles A, Kastrati I (2022). Selenium and breast cancer: an update of clinical and epidemiological data. Arch Biochem Biophys.

[4] Demircan K, Sun Q, Bengtsson Y, Seemann P, Vallon-Christersson J, Malmberg M (2022). Autoimmunity to selenoprotein P predicts breast cancer recurrence. Redox Biol.

[5] Demircan K, Bengtsson Y, Sun Q, Brange A, Vallon-Christersson J, Rijntjes E (2021). Serum selenium, selenoprotein P and glutathione peroxidase 3 as predictors of mortality and recurrence following breast cancer diagnosis: a multicentre cohort study. Redox Biol.

[6] Sandsveden M, Nilsson E, Borgquist S, Rosendahl AH, Manjer J (2020). Prediagnostic serum selenium levels in relation to breast cancer survival and tumor characteristics. Int J Cancer.

[7] Lubinski J, Marciniak W, Muszynska M, Huzarski T, Gronwald J, Cybulski C (2018). Serum selenium levels predict survival after breast cancer. Breast Cancer Res Treat.

[8] Harris HR, Bergkvist L, Wolk A (2012). Selenium intake and breast cancer mortality in a cohort of swedish women. Breast Cancer Res Treat.

[9] Labunskyy VM, Hatfield DL, Gladyshev VN (2014). Selenoproteins: molecular pathways and physiological roles. Physiol Rev.

[10] Schomburg L (2012). Selenium, selenoproteins and the thyroid gland: interactions in health and disease. Nat Rev Endocrinol.

[11] Steinbrenner H, Speckmann B, Klotz LO (2016). Selenoproteins: antioxidant selenoenzymes and beyond. Arch Biochem Biophys.

[12] Xia Y, Hill KE, Li P, Xu J, Zhou D, Motley AK (2010). Optimization of selenoprotein P and other plasma selenium biomarkers for the assessment of the selenium nutritional requirement: a placebo-controlled, double-blind study of selenomethionine supplementation in selenium-deficient chinese subjects. Am J Clin Nutr.

[13] Hoffmann PR, Höge SC, Li PA, Hoffmann FW, Hashimoto AC, Berry MJ (2007). The selenoproteome exhibits widely varying, tissue-specific dependence on selenoprotein P for selenium supply. Nucleic Acids Res.

[14] Goemann IM, Marczyk VR, Recamonde-Mendoza M, Wajner SM, Graudenz MS, Maia AL (2020). Decreased expression of the thyroid hormone-inactivating enzyme type 3 deiodinase is associated with lower survival rates in breast cancer. Sci Rep.

[15] Lou W, Ding B, Wang S, Fu P (2020). Overexpression of GPX3, a potential biomarker for diagnosis and prognosis of breast cancer, inhibits progression of breast cancer cells in vitro. Cancer Cell Int.

[16] Cadenas C, Franckenstein D, Schmidt M, Gehrmann M, Hermes M, Geppert B (2010). Role of thioredoxin reductase 1 and thioredoxin interacting protein in prognosis of breast cancer. Breast Cancer Res.

[17] Li Z, Ferguson L, Deol KK, Roberts MA, Magtanong L, Hendricks JM (2022). Ribosome stalling during selenoprotein translation exposes a ferroptosis vulnerability. Nat Chem Biol.

[18] Hilal T, Killam BY, Grozdanović M, Dobosz-Bartoszek M, Loerke J, Bürger J (2022). Structure of the mammalian ribosome as it decodes the selenocysteine UGA codon. Science.

[19] Saal LH, Vallon-Christersson J, Häkkinen J, Hegardt C, Grabau D, Winter C (2015). The Sweden Cancerome Analysis network-breast (SCAN-B) Initiative: a large-scale multicenter infrastructure towards implementation of breast cancer genomic analyses in the clinical routine. Genome Med.

[20] Löfgren L, Eloranta S, Krawiec K, Asterkvist A, Lönnqvist C, Sandelin K (2019). Validation of data quality in the Swedish National Register for breast cancer. BMC Public Health.

[21] Staaf J, Häkkinen J, Hegardt C, Saal LH, Kimbung S, Hedenfalk I (2022). RNA sequencing-based single sample predictors of molecular subtype and risk of recurrence for clinical assessment of early-stage breast cancer. NPJ Breast Cancer.

[22] Wu SZ, Al-Eryani G, Roden DL, Junankar S, Harvey K, Andersson A (2021). A single-cell and spatially resolved atlas of human breast cancers. Nat Genet.

[23] Nappi A, De Stefano MA, Dentice M, Salvatore D (2021). Deiodinases and cancer. Endocrinology.

[24] Goemann IM, Marczyk VR, Romitti M, Wajner SM, Maia AL (2018). Current concepts and challenges to unravel the role of iodothyronine deiodinases in human neoplasias. Endocr Relat Cancer.

[25] Robinson DR, Wu YM, Vats P, Su F, Lonigro RJ, Cao X (2013). Activating ESR1 mutations in hormone-resistant metastatic breast cancer. Nat Genet.

[26] Bose R, Kavuri SM, Searleman AC, Shen W, Shen D, Koboldt DC (2013). Activating HER2 mutations in HER2 gene amplification negative breast cancer. Cancer Discov.

[27] Brueffer C, Vallon-Christersson J, Grabau D, Ehinger A, Häkkinen J, Hegardt C et al. Clinical value of RNA sequencing–based classifiers for prediction of the five conventional breast Cancer biomarkers: a Report from the Population-Based Multicenter Sweden Cancerome Analysis Network—Breast Initiative. JCO Precis Oncol. 2018;(2):1–18. 10.1200/PO.17.00135 PMC7446376 32913985

[28] Sotiriou C, Pusztai L (2009). Gene-expression signatures in breast cancer. N Engl J Med.

[29] Reis-Filho JS, Pusztai L (2011). Gene expression profiling in breast cancer: classification, prognostication, and prediction. Lancet.

[30] Driscoll DM, Copeland PR, MECHANISM AND REGULATION OF SELENOPROTEIN SYNTHESIS (2003). Annu Rev Nutr.

[31] Turanov AA, Everley RA, Hybsier S, Renko K, Schomburg L, Gygi SP (2015). Regulation of selenocysteine content of human selenoprotein P by dietary selenium and insertion of cysteine in place of selenocysteine. PLoS ONE.

[32] Xia Y, Hill KE, Byrne DW, Xu J, Burk RF (2005). Effectiveness of selenium supplements in a low-selenium area of China2. Am J Clin Nutr.

[33] Köhrle J, Frädrich C (2022). Deiodinases control local cellular and systemic thyroid hormone availability. Free Radic Biol Med.

[34] Krashin E, Silverman B, Steinberg DM, Yekutieli D, Giveon S, Fabian O (2021). Pre-diagnosis thyroid hormone dysfunction is associated with cancer mortality. Endocr Relat Cancer.

[35] Sandsveden M, Borgquist S, Rosendahl AH, Manjer J (2021). Low thyroid hormone receptor alpha-2 (THRα-2) tumor expression is associated with unfavorable tumor characteristics and high breast cancer mortality. Breast Cancer Res.

[36] Journy NMY, Bernier MO, Doody MM, Alexander BH, Linet MS, Kitahara CM (2017). Hyperthyroidism, hypothyroidism, and cause-specific mortality in a large cohort of women. Thyroid.

[37] Brandt J, Borgquist S, Almquist M, Manjer J (2016). Thyroid function and survival following breast cancer. BJS (Br J Surg).

[38] Tosovic A, Bondeson A-G, Bondeson L, Ericsson U-B, Manjer J (2013). Triiodothyronine levels in relation to mortality from breast cancer and all causes: a population-based prospective cohort study. Eur J Endocrinol.

[39] Pascual A, Aranda A (2013). Thyroid hormone receptors, cell growth and differentiation. Biochim Biophys Acta.

[40] Kowalik MA, Puliga E, Cabras L, Sulas P, Petrelli A, Perra A (2020). Thyroid hormone inhibits hepatocellular carcinoma progression via induction of differentiation and metabolic reprogramming. J Hepatol.

[41] Dentice M, Luongo C, Huang S, Ambrosio R, Elefante A, Mirebeau-Prunier D (2007). Sonic hedgehog-induced type 3 deiodinase blocks thyroid hormone action enhancing proliferation of normal and malignant keratinocytes. Proc Natl Acad Sci..

[42] Labunskyy VM, Hatfield DL, Gladyshev VN (2007). The Sep15 protein family: roles in disulfide bond formation and quality control in the endoplasmic reticulum. IUBMB Life.

[43] Ferguson AD, Labunskyy VM, Fomenko DE, Araç D, Chelliah Y, Amezcua CA (2006). NMR structures of the selenoproteins Sep15 and SelM reveal redox activity of a new thioredoxin-like family*. J Biol Chem.

[44] Davis CD, Tsuji PA, Milner JA (2012). Selenoproteins and cancer prevention. Annu Rev Nutr.

[45] Zigrossi A, Hong LK, Ekyalongo RC, Cruz-Alvarez C, Gornick E, Diamond AM (2022). SELENOF is a new tumor suppressor in breast cancer. Oncogene.

[46] Lan X, Xing J, Gao H, Li S, Quan L, Jiang Y (2017). Decreased expression of Selenoproteins as a poor prognosticator of gastric cancer in humans. Biol Trace Elem Res.

[47] Dai X, Thongchot S, Dokduang H, Loilome W, Khuntikeo N, Titapun A (2016). Potential of Selenium Compounds as New Anticancer Agents for Cholangiocarcinoma. Anticancer Res.

[48] Schomburg L, Schweizer U (2009). Hierarchical regulation of selenoprotein expression and sex-specific effects of selenium. Biochim Biophys Acta (BBA) Gen Subj..

[49] Bermano G, Nicol F, Dyer JA, Sunde RA, Beckett GJ, Arthur JR (1995). Tissue-specific regulation of selenoenzyme gene expression during selenium deficiency in rats. Biochem J.

[50] Baker JR, Umesh S, Jenab M, Schomburg L, Tjønneland A, Olsen A (2021). Prediagnostic blood selenium status and mortality among patients with colorectal cancer in western european populations. Biomedicines.

[51] Bleys J, Navas-Acien A, Guallar E (2008). Serum selenium levels and all-cause, cancer, and cardiovascular mortality among US adults. Arch Intern Med.

[52] Rogoża-Janiszewska E, Malińska K, Baszuk P, Marciniak W, Derkacz R, Lener M (2021). Serum selenium level and 10-year survival after melanoma. Biomedicines.

[53] Lubiński J, Marciniak W, Muszynska M, Jaworowska E, Sulikowski M, Jakubowska A (2018). Serum selenium levels and the risk of progression of laryngeal cancer. PLoS ONE.

[54] Pietrzak S, Wójcik J, Scott RJ, Kashyap A, Grodzki T, Baszuk P (2019). Influence of the selenium level on overall survival in lung cancer. J Trace Elem Med Biol.

[55] Vallon-Christersson JRNA. Sequencing-based single sample predictors of molecular subtype and risk of recurrence for clinical assessment of early-stage breast cancer. 3 edn. Mendeley Data 2023. 10.1038/s41523-022-00465-3 PMC9381586 35974007

