# Supplementary material for: Matched analysis of circulating selenium with the breast cancer selenotranscriptome: a multicentre prospective study
Source: J Transl Med. 2023 Sep 23;21:658. doi: 10.1186/s12967-023-04502-y (PMC10517476; doi:10.1186/s12967-023-04502-y)
Supplement: Supplementary file 1 — Additional file 1: Figure S1. Missing values in variables used in Cox regression models. Figure S2. Study flow chart. Adapted from Demircan K, Bengtsson Y, Sun Q, Brange A, Vallon-Christersson J, Rijntjes E, Malmberg M, Saal LH, Rydén L, Borg Å, Manjer J, Schomburg L. Serum selenium, selenoprotein P and glutathione peroxidase 3 as predictors of mortality and recurrence following breast cancer diagnosis: A multicentre cohort study. Redox Biol. 2021 Nov;47:102145. Figure S3. Cox regression models in the whole cohort and in low and high SELENOP subgroups. Subgroups were divided according to median SELENOP concentration of the cohort, i.e. 4.05 mg/L. All models were adjusted for age, tumour size, histological grade, lymph node involvement, expression of HER2/ER/PGR-Receptor, laterality of the tumour, and histological type. P for interaction was tested by adding an interaction term between serum SELENOP and the gene of interest, marked by purple asterisk. Figure S4. Cox regression models in the whole cohort and in low and high GPx3 subgroups. Subgroups were divided according to median GPx3 concentration of the cohort, i.e. 205 U/L. All models were adjusted for age, tumour size, histological grade, lymph node involvement, expression of HER2/ER/PGR-Receptor, laterality of the tumour, and histological type. P for interaction was tested by adding an interaction term between serum GPx3 and the gene of interest, marked by purple asterisk. Figure S5. Kaplan Meier analyses of genes interacting with serum selenium in TCGA-BRCA data. Patients were compared according to mRNA expression for each candidate gene, based on being in the highest quartile (Q4) vs lowest (Q1). Log-rank test was applied to detect differences. GEPIA2 was used to plot survival, accessed on 25th August 2023, on http://gepia2.cancer-pku.cn/). Tang, Z. et al. (2019) GEPIA2: an enhanced web server for large-scale expression profiling and interactive analysis. Nucleic Acids Res, 10.1093/nar/gkz430. Table S1. P val [file 12967_2023_4502_MOESM1_ESM.docx]

**Additional file**

**for**

**Matched analysis of circulating selenium with the breast cancer selenotranscriptome: a multicentre prospective study**

Kamil Demircan**^1,2^**, Ylva Bengtsson**^3^**, Thilo Samson Chillon**^1^**, Johan Vallon-Christersson**^4^**, Qian Sun**^1^**, Christer Larsson**^5^**, Martin Malmberg**^6^**, Lao H Saal**^4^**, Lisa Rydén**^3^**, Åke Borg**^4^**, Jonas Manjer**^3,^*** and Lutz Schomburg**^1,^***

**
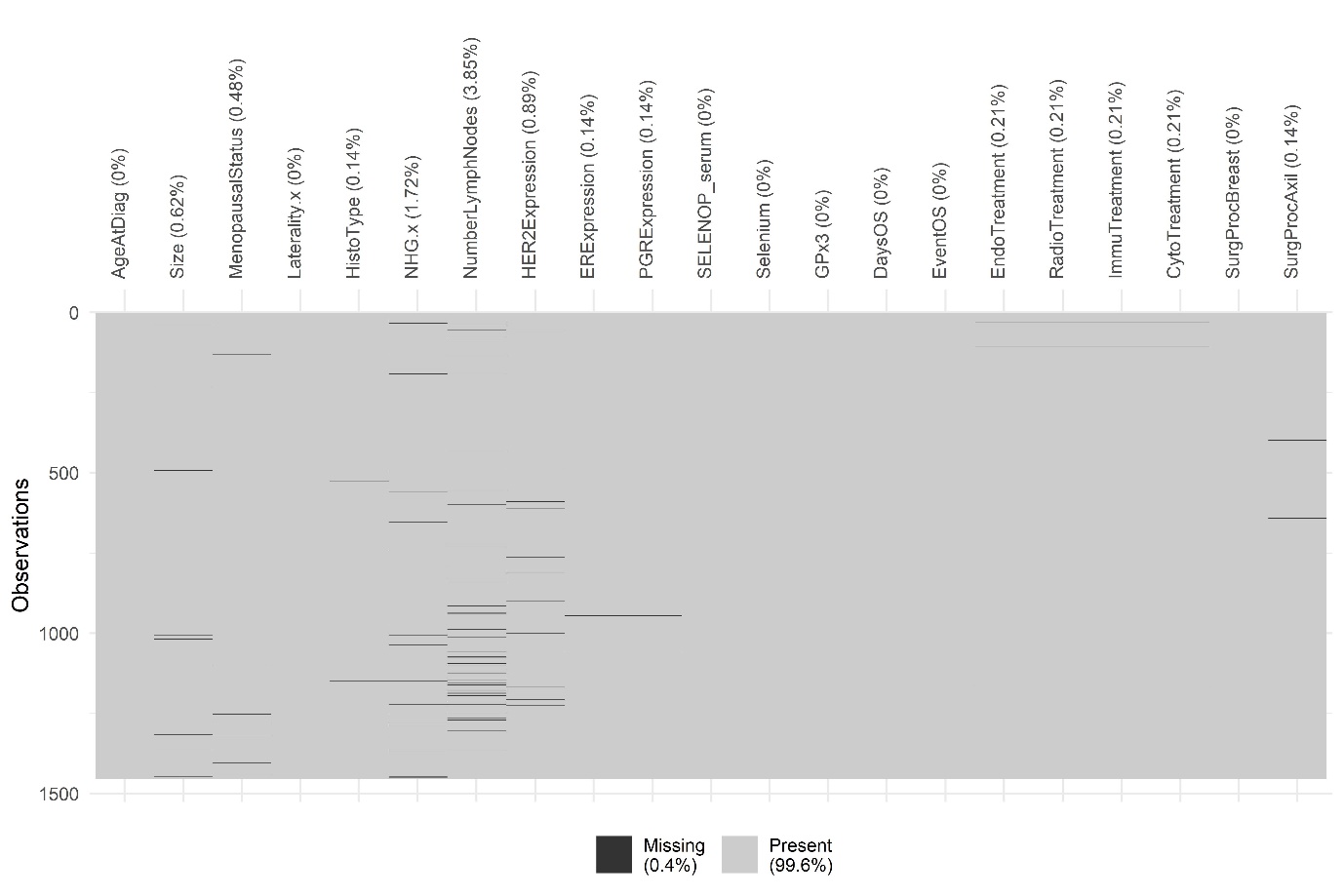
**

**Figure S1.** Missing values in variables used in Cox regression models.


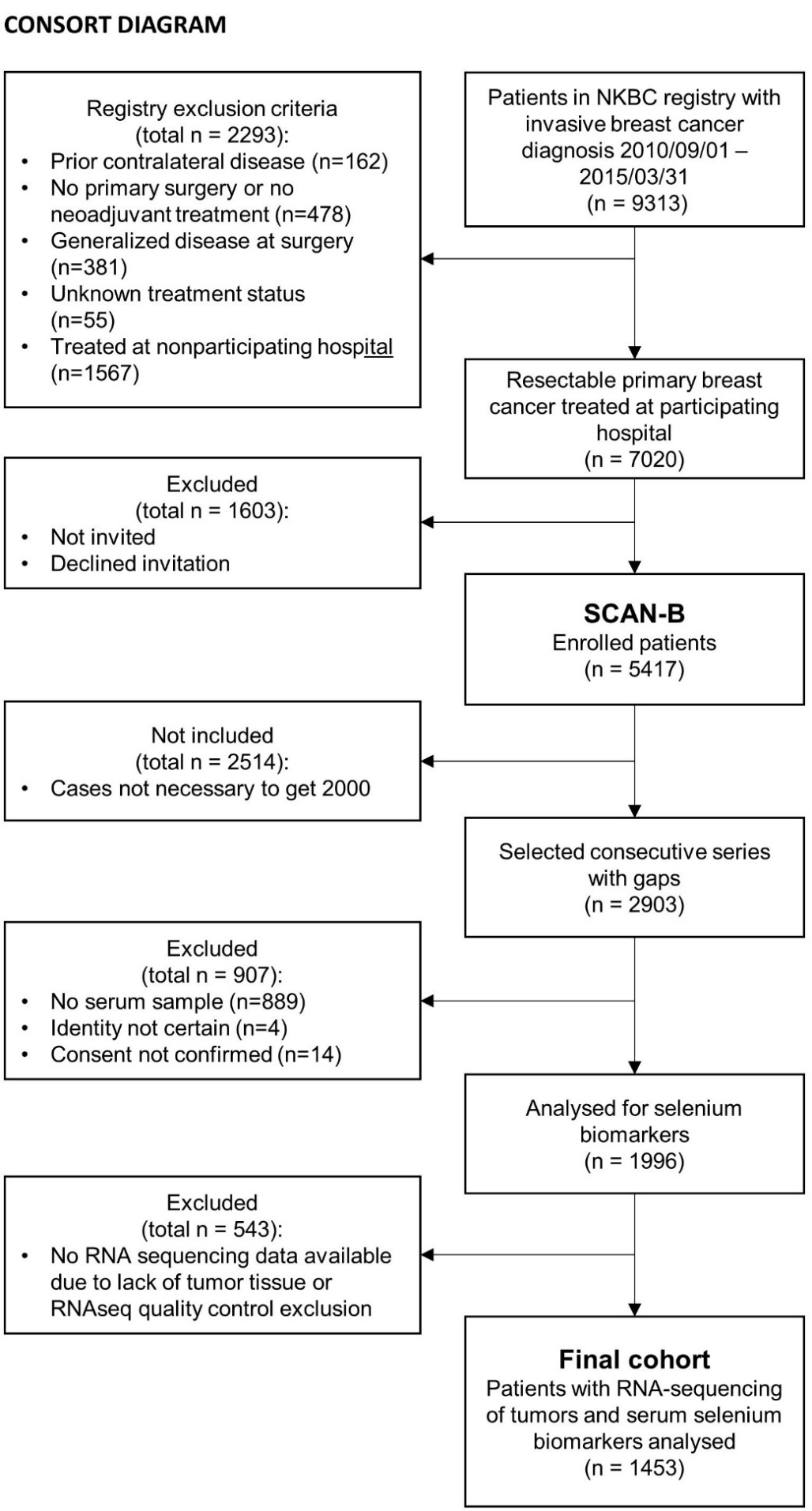


**Figure S2.** Study flow chart. Adapted from Demircan K, Bengtsson Y, Sun Q, Brange A, Vallon-Christersson J, Rijntjes E, Malmberg M, Saal LH, Rydén L, Borg Å, Manjer J, Schomburg L. Serum selenium, selenoprotein P and glutathione peroxidase 3 as predictors of mortality and recurrence following breast cancer diagnosis: A multicentre cohort study. Redox Biol. 2021 Nov;47:102145.

**
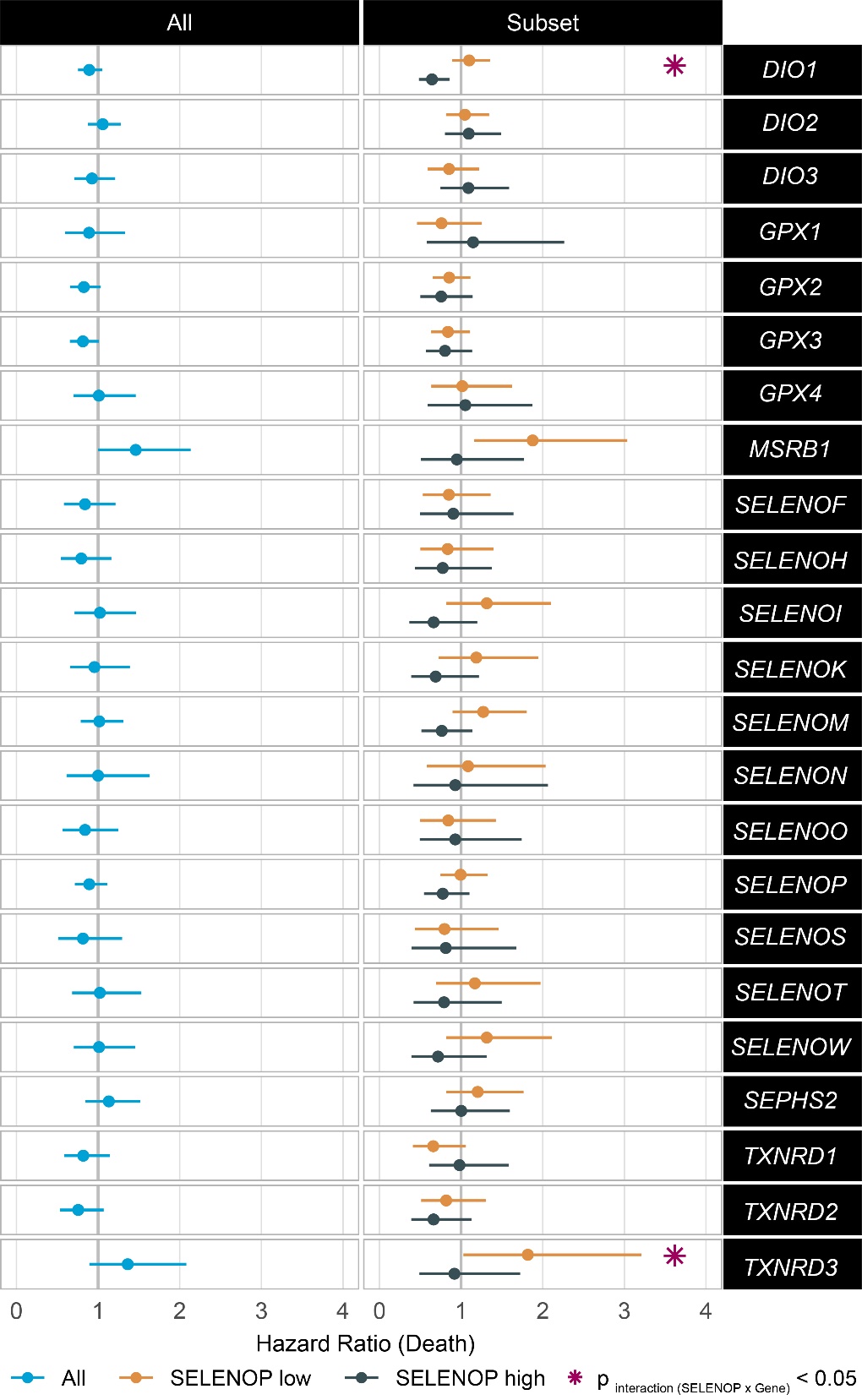
**

**Figure S3.** Cox regression models in the whole cohort and in low and high SELENOP subgroups. Subgroups were divided according to median SELENOP concentration of the cohort, i.e. 4.05 mg/L. All models were adjusted for age, tumour size, histological grade, lymph node involvement, expression of HER2/ER/PGR-Receptor, laterality of the tumour, and histological type. P for interaction was tested by adding an interaction term between serum SELENOP and the gene of interest, marked by purple asterisk.

**
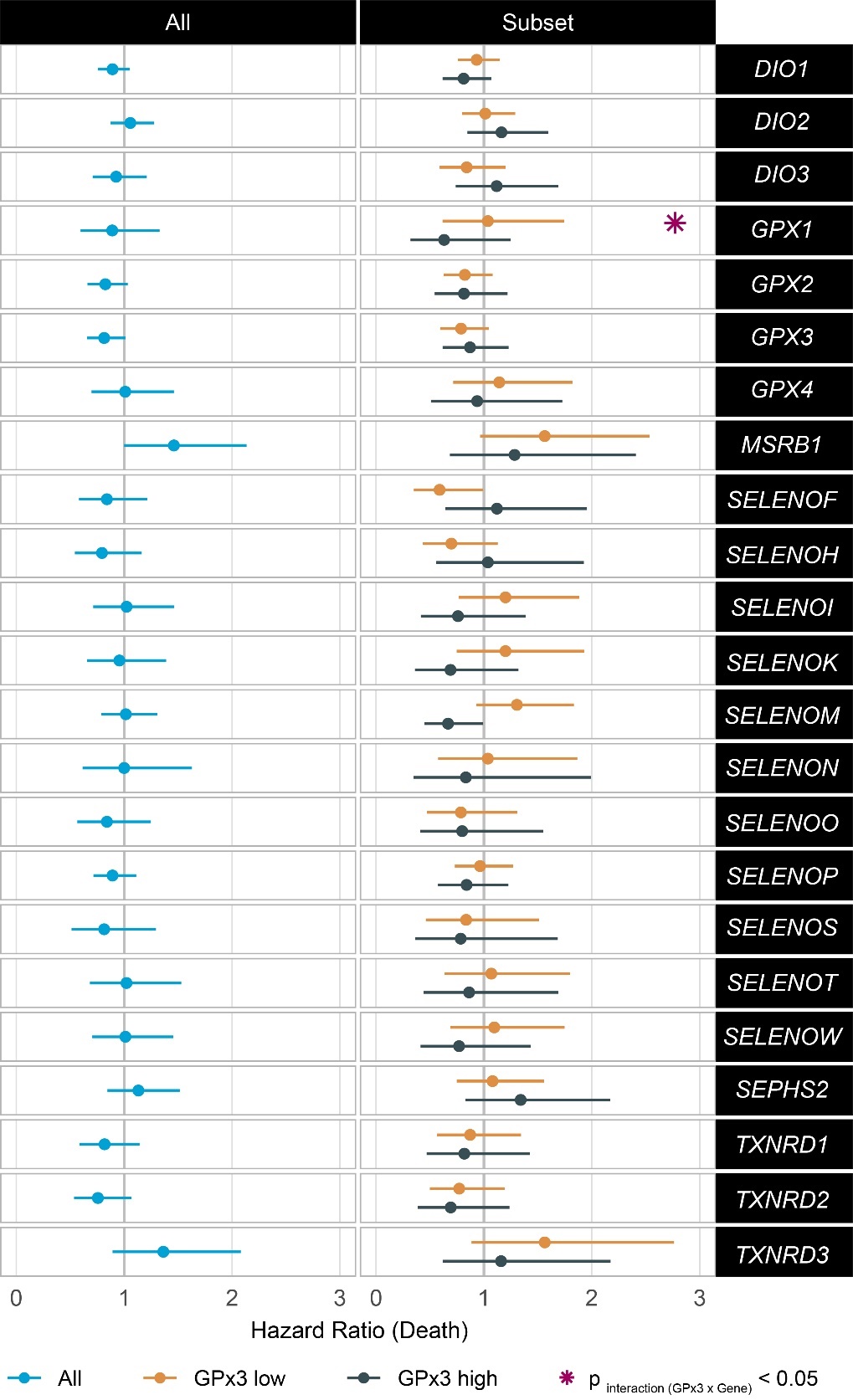
**

**Figure S4.** Cox regression models in the whole cohort and in low and high GPx3 subgroups. Subgroups were divided according to median GPx3 concentration of the cohort, i.e. 205 U/L. All models were adjusted for age, tumour size, histological grade, lymph node involvement, expression of HER2/ER/PGR-Receptor, laterality of the tumour, and histological type. P for interaction was tested by adding an interaction term between serum GPx3 and the gene of interest, marked by purple asterisk.

**
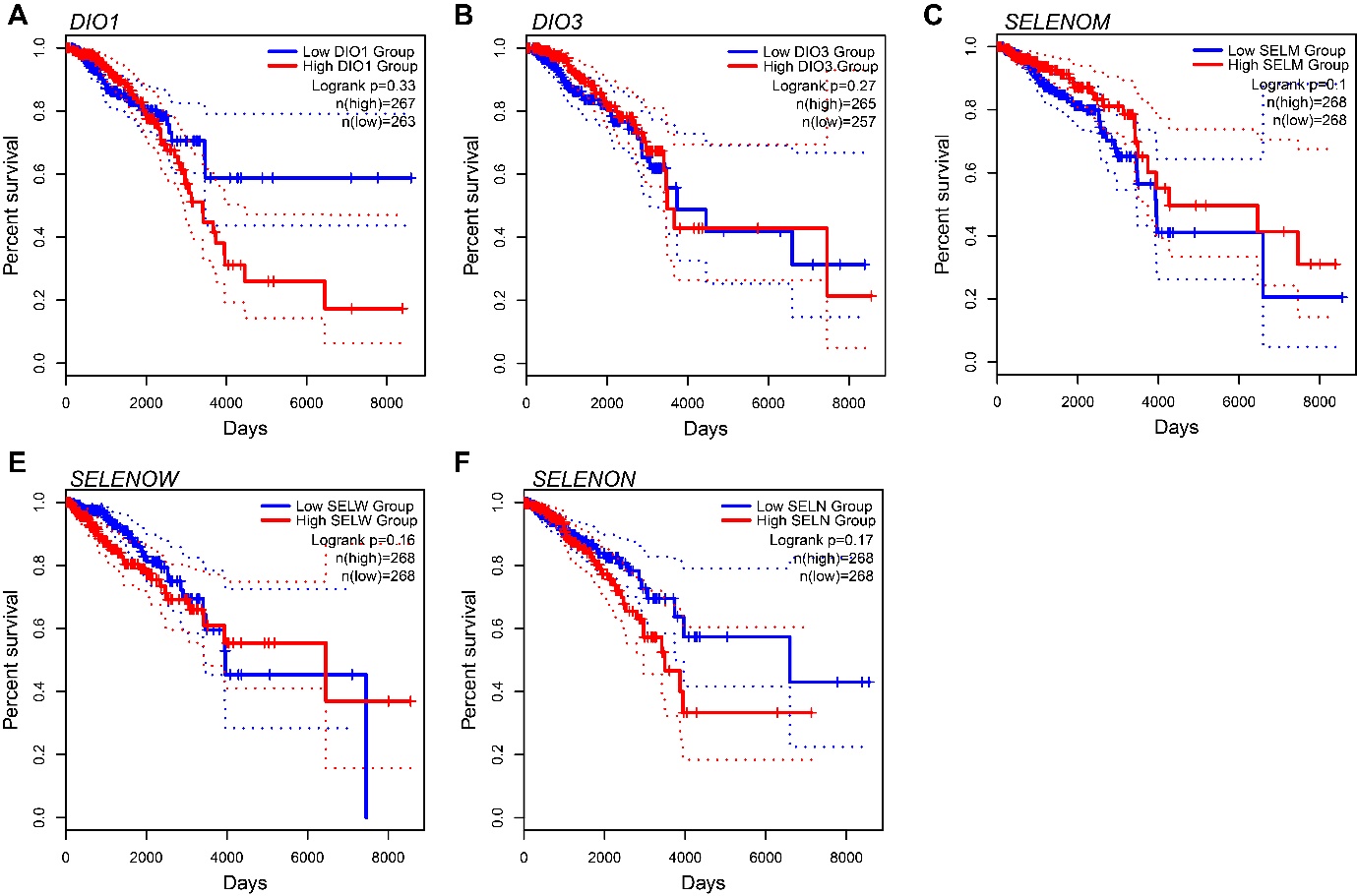
**

**Figure S5.** Kaplan Meier analyses of genes interacting with serum selenium in TCGA-BRCA data. Patients were compared according to mRNA expression for each candidate gene, based on being in the highest quartile (Q4) vs lowest (Q1). Log-rank test was applied to detect differences. GEPIA2 was used to plot survival, accessed on 25th August 2023, on http://gepia2.cancer-pku.cn/). Tang, Z. et al. (2019) GEPIA2: an enhanced web server for large-scale expression profiling and interactive analysis. Nucleic Acids Res, 10.1093/nar/gkz430.

**Table S1**. P values for interaction between DIO1, DIO3, SELENOM and serum selenium, further adjusted for therapy regimens

|  | Genes | Fully adjusted* | +Endocrine  Treatment | +Immune  Treatment | +Chemo-therapy | +Radio-  treatment | +Surgery  Breast | +Surgery  Axilla | All treatment |
| --- | --- | --- | --- | --- | --- | --- | --- | --- | --- |
| p_interaction_ | *DIO1* | <0.001 | <0.001 | <0.001 | <0.001 | <0.001 | <0.001 | 0.001 | <0.001 |
|  | *DIO3* | 0.020 | 0.022 | 0.047 | 0.023 | 0.024 | 0.020 | 0.017 | 0.041 |
|  | *SELENOM* | 0.038 | 0.038 | 0.044 | 0.037 | 0.041 | 0.031 | 0.045 | 0.038 |

* Adjusted for age at diagnosis, tumour size, laterality of the tumour, Nottingham histological grade, lymph node involvement, HER2R expression, ER expression PGR expression and histological type.
